# Increased Autophagy Levels Mediate Cisplatin Resistance in Cisplatin-Resistant Cells While Also Rendering Them Vulnerable to Autophagy Induction

**DOI:** 10.1155/2018/1736738

**Published:** 2018-11-12

**Authors:** Guihua Duan, Zhengji Song, Min Qi, Xuan Bai, Jingzhai Wang, Yu Zhang, Xiaoping Zou, Qiang Guo, Ping Wan

**Affiliations:** ^1^Department of Gastroenterology, The First People's Hospital of Yunnan Province, The Affiliated Hospital of Kunming University of Science and Technology, Kunming 650032, China; ^2^Department of Gastroenterology, Drum Tower Hospital, Medical School of Nanjing University, Nanjing 210008, China; ^3^Department of Radiology, The First People's Hospital of Yunnan Province, The Affiliated Hospital of Kunming University of Science and Technology, Kunming 650032, China

## Abstract

Autophagy plays an important role in tumor development because of its capacity to maintain energy homeostasis by recycling damaged intracellular proteins and organelles, and increased autophagy levels are reported to mediate drug resistance in many cancers. However, whether high autophagy levels negatively impact tumor cell growth is unknown. Herein, we found that cisplatin (ddp)-resistant cells were more sensitive to glutamine (Gln) deprivation than ddp-sensitive cells, and they showed significant G1 arrest and increased apoptosis rates under Gln-deficient conditions. Furthermore, ddp-resistant cells had a higher level of autophagy, which mediated ddp resistance. Further analysis indicated that Gln deficiency could trigger apoptosis by enhancing activation of the autophagy signaling pathway AMPK/ULK1 in ddp-resistant cells due to their high basal autophagy level. Interestingly, ddp-resistant cells were more sensitive to rapamycin, and rapamycin could efficiently suppress the growth of ddp-resistant cells* in vivo*. Taken together, our study demonstrated that ddp-resistant cells became vulnerable to Gln deprivation because of their increased level of autophagy, and for the first time, we showed that suppressing the growth of ddp-resistant cells via enhancing autophagy induction was possible with rapamycin treatment.

## 1. Introduction

Cisplatin (ddp) is a largely employed platinum-based drug that exerts clinical activity against a wide range of solid tumors [1]. Although ddp treatment is often accompanied by initial therapeutic success associated with partial responses or disease stabilization, the development of chemoresistance inevitably leads to therapeutic failure [1], and strategies for overcoming ddp resistance remain a current challenge.

Autophagy is an evolutionarily conserved catabolic process by which misfolded proteins and damaged organelles are degraded in lysosomes [2], and its main role is to maintain energy homeostasis by recycling altered and damaged intracellular proteins and organelles, thus promoting cell survival [3, 4]. Autophagy plays pivotal roles in the quality control of cellular structural components and provides materials and nutrients for newly constructed structures in cells under several stresses, including metabolic and oxidative stress, and occurs in both normal and tumor cells [3, 4]. Autophagy also plays an important role in tumor treatment. Acute ddp treatment can induce autophagy, which serves as a survival factor to protect cells from ddp-induced cell death [1]. Autophagy has been demonstrated to contribute to ddp resistance in esophageal [5], cervical [6], ovarian [7], prostate [8], gastric [9], osteosarcoma [10], and head and neck [11] cancers, and autophagy inhibition can reduce ddp resistance to some extent. Studies have indicated that although drug resistance mechanisms may help tumor cells tolerate cytotoxic treatments, cellular resistance adaptability functions as a double-edged sword because it comes with a fitness cost [12–15]. The impact of increased autophagic response on cell adaptability in ddp-resistant cells remains unelucidated.

Glutamine (Gln), the most abundant amino acid in the blood, plays a particularly important role in cell growth and metabolism. Gln is metabolized via a process termed glutaminolysis, which can suppress autophagy via different mechanisms [16, 17]. Whether glutaminolysis has diverse impacts on the proliferation of ddp-sensitive cells and ddp-resistant cells by affecting the autophagic response has not been determined.

In this study, we demonstrated that the growth of ddp-resistant cells was dependent on Gln, and Gln deprivation induced cell cycle arrest and apoptosis in ddp-resistant cells. Further analysis indicated that ddp-resistant cells had a higher autophagy level than ddp-sensitive cells, which mediated ddp resistance. Based on this trait, Gln deprivation induces autophagy in ddp-sensitive cells, promoting stress survival, but triggers apoptosis by enhancing activation of the autophagy signaling pathway in ddp-resistant cells. Meanwhile, ddp-resistant cells were found to be more sensitive to an autophagy inducer, rapamycin, and rapamycin could effectively inhibit the growth of ddp-resistant tumors in a xenograft model. Our results indicated that increased autophagy levels could help ddp-resistant cells maintain drug resistance but could also serve as a potential therapeutic target.

## 2. Materials and Methods

Our study protocol was approved by the Ethics Review Committee for Animal Experimentation at the First People's Hospital of Yunnan Province (Kunming, China). All animal procedures were performed in compliance with guidelines set by the Animal Care Committee, and all efforts were made to reduce possible pain and discomfort of the animals.

### 2.1. Cell Culture

HeLa (cervical cancer cell line), HGC27 (gastric cancer cell line), and AGS (gastric cancer cell line) cells were purchased from the Type Culture Collection of the Chinese Academy of Sciences in Shanghai, China. The HeLa/ddp, HGC27/ddp, and AGS/ddp cell lines were established by exposure of the parental cells to gradually increasing concentrations of ddp (Sigma) as described in our previous study [15]. HeLa-GFP-LC3 and HeLa/ddp-GFP-LC3 cells were derived via the lentiviral transduction of HeLa and HeLa/ddp cells with TagGFP2-LC3 lentivirus (Millipore). All cell lines were authenticated by short tandem repeat profiling analysis performed in 2014. All cell lines were cultured in RPMI 1640 (Invitrogen) medium supplemented with 10% FBS (Biological Industries, BI) and 1% penicillin/streptomycin (50 *μ*nits/ml, 50 *μ*g/ml, Invitrogen) at a 5% CO_2_. All media used in our experiments were supplemented with 10% FBS and 1% penicillin/streptomycin.

### 2.2. Cell Growth Analysis

For cell number assays, cells were plated in 24-well plates at 1×10^5^ (HeLa) or 5×10^4^ (HGC27 and AGS) cells per well in 0.5 ml of media. For Gln deprivation, cells were initially plated in complete culture media (2 mM Gln), which was replaced with Gln-free medium the following day. The medium was not changed throughout the remainder of the experiment. At the indicated time points, cells in triplicate wells were trypsinized and counted with a hand-held automated cell counter (Scepter 2.0, Millipore).

For relative cell number assays, cells were plated in 24-well plates at 1.5×10^4^ cells per well in 0.5 ml of media. On the following day, the medium was replaced with medium containing the desired inhibitors. After 3 days, cells were fixed in 10% formalin and stained with 0.1% crystal violet. The dye was extracted with 10% acetic acid, and relative proliferation was measured by absorbance at 490 nm.

### 2.3. Colony-Formation Assays

Cells were plated in 6-well plates at 500-1,000 cells per well in 2 ml of media. The medium was not changed throughout the course of the experiment. After 10-14 days, colonies were fixed in methanol and stained with 0.5% crystal violet. Colonies of more than 50 cells were counted under a microscope.

### 2.4. EdU Assay

The EdU assay was performed using the Click-iT® Plus EdU Imaging Kit (Invitrogen) according to the manufacturer's instructions. The percentage of EdU^+^ cells was determined by counting an average of 500-1,000 cells per field from 3 randomly selected sample regions using ImageJ software.

### 2.5. Analysis of Apoptosis and Cell Cycle Distribution

Apoptosis rates and cell cycle analysis* in vitro* were performed using the Annexin V-FITC Apoptosis Detection Kit (Miltenyi Biotec) and Cycletest™ Plus DNA Reagent Kit (BD Bioscience), respectively, according to the manufacturers' instructions. Cells were analyzed by flow cytometry to determine their apoptosis rates and cell cycle distributions.

### 2.6. ROS Quantification

Cells were incubated with 5 *μ*M 2′,7′-dichlorofluorescin diacetate (DCFDA, Sigma) for 25 min and washed twice with PBS, and labeled cells were then trypsinized and resuspended in PBS. Mean fluorescence intensity was analyzed by flow cytometry.

### 2.7. Autophagy Measurements

HeLa-GFP-LC3 and HeLa/ddp-GFP-LC3 cells were seeded in 12-well plates at 20,000 cells per well. The medium was exchanged for indicated medium the following day, and GFP-LC3 puncta were detected after incubation 24h using a fluorescence microscope (Leica). Punctate areas per cell were analyzed in three images containing more than 15 cells using ImageJ software.

### 2.8. Western Blot Analysis

Cells were washed and lysed in RIPA buffer supplemented with protease/phosphatase inhibitor cocktail (5872, Cell Signaling Technology, CST). Protein lysates were separated by SDS-PAGE and transferred to polyvinylidene fluoride membranes. The membranes were blocked in TBST (Tris-buffered saline containing 0.1% Tween-20) containing 5% nonfat dry milk or bovine serum albumin for 2 h before incubation with the desired primary antibodies at dilutions recommended by the manufacturers overnight. Then, the membranes were incubated with the appropriate horseradish peroxidase (HRP)-conjugated secondary antibody (1:5000 dilutions). Signals generated by enhanced chemiluminescence (Millipore) were recorded with a charge-coupled device (CCD) camera (CLINX, Shanghai, China). Data are representative of at least three independent experiments. The following antibodies were used: LC3 (12741, CST), AMPK (5831, CST), p-AMPK (2535, CST), ULK1 (6439, CST), p-ULK1 (12753, CST), cleaved caspase-3 (9661, CST), caspase-3 (9662, CST), and *β*-actin (A5441, Sigma).

### 2.9. Xenograft Experiments

All animal procedures were performed in compliance with guidelines set by the Animal Care Committee of the First People's Hospital of Yunnan Province (Kunming, China). Male Nu/Nu mice were purchased from Vital River Laboratories. For subcutaneous xenografts, 2×10^6^ cells suspended in 0.1 ml of 50% Matrigel (BD Biosciences) solution in RPMI 1640 medium were injected subcutaneously into the lower flanks of 4-week-old Nu/Nu mice. Tumor lengths and widths were measured every 2–3 days with a caliper, and tumor volumes were calculated according to the formula 1/2×length×width^2^. Mice with established tumors (50-100 mm^3^) were randomized and received treatment with rapamycin (2 mg/kg) or solvent by intraperitoneal (i.p.) injection daily for 14 days. Rapamycin was dissolved in solvent solution (0.2% carboxymethylcellulose and 0.25% Tween-80 in sterile H_2_O).

### 2.10. Statistical Analyses

All experiments were performed in at least triplicate, and data are reported as the mean ± SD. Differences between groups were evaluated by ANOVA or Student's t-tests. Differences were considered significant if P<0.05 (*∗*, P<0.05; *∗∗*, P<0.01; and *∗∗∗*, P<0.001). All statistical analyses were performed using GraphPad Prism software (6.0).

## 3. Results

### 3.1. Ddp-Resistant Cells Rely on Gln More for Growth Than ddp-Sensitive Cells

**Figure 1:**
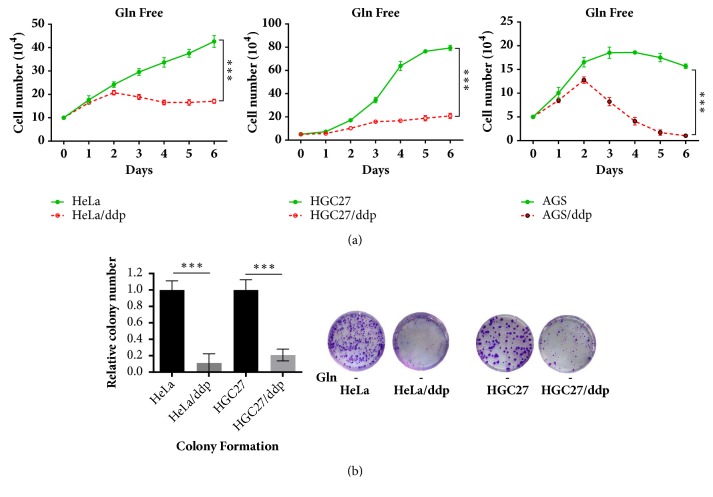
Ddp-resistant cells are more dependent on Gln for growth than ddp-sensitive cells. (a) Growth of HeLa, HeLa/ddp, HGC27, HGC27/ddp, AGS, and AGS/ddp cells under Gln-deficient conditions; the medium was exchanged for Gln-free medium the day after seeding. (b) Relative clonogenic growth of HeLa, HeLa/ddp, HGC27, and HGC27/ddp cells under Gln-deficient conditions; the medium was exchanged for Gln-free medium the day after seeding. The error bars represent the s.d. of triplicate wells of a representative experiment; *∗∗∗*P<0.001.

**Figure 2:**
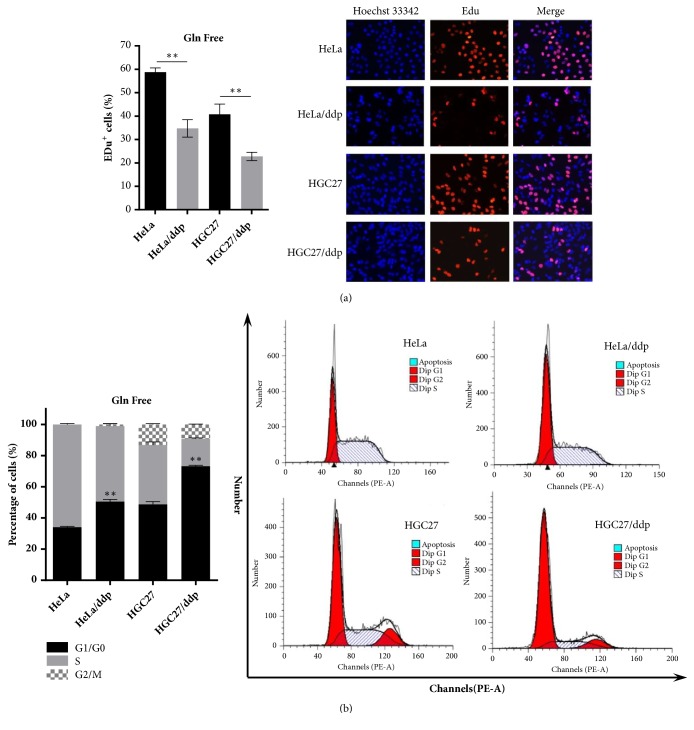
Gln deprivation reduces DNA synthesis in ddp-resistant cells. (a) EdU assays of HeLa, HeLa/ddp, HGC27, and HGC27/ddp cells. The medium was exchanged for Gln-free medium the day after seeding, and EdU assays were performed 24 h later. (b) Cell cycle analysis of HeLa, HeLa/ddp, HGC27, and HGC27/ddp cells. The medium was exchanged for Gln-free medium the day after seeding, and cell cycle distribution was analyzed 24 h later. The error bars represent the s.d. of triplicate wells of a representative experiment; *∗∗*P<0.01.

**Figure 3:**
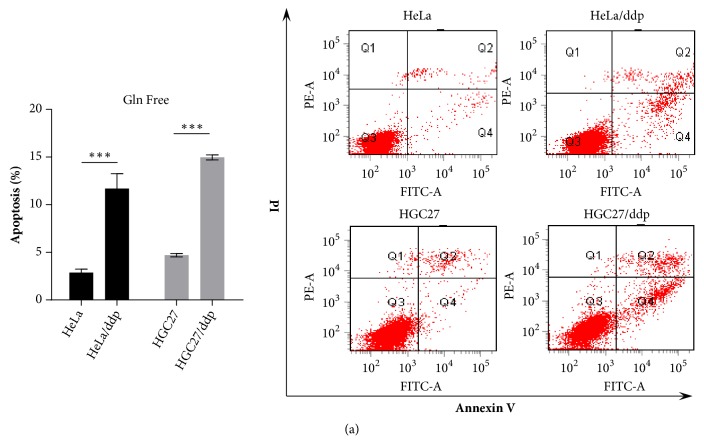
Gln deprivation increases apoptosis in ddp-resistant cells. (a) Apoptosis of HeLa, HeLa/ddp, HGC27, and HGC27/ddp cells under Gln-deficient conditions. The medium was exchanged for Gln-free medium the day after seeding, and apoptosis rates were analyzed 72 h later. The error bars represent the s.d. of triplicate wells of a representative experiment; *∗∗∗*P<0.001.

First, we investigated the impact of Gln on the growth of both cell lines. HeLa/ddp and HGC27/ddp cells showed growth arrest, and AGS/ddp cells showed growth regression in Gln-free conditions compared with the corresponding sensitive cells, suggesting that ddp-resistant cells are extremely addicted to Gln (Figures 1(a) and 1(b)). Cell cycle analysis and EdU incorporation assays indicated significant G1 arrest and reduced DNA synthesis in both HeLa/ddp and HGC27/ddp cells in Gln-free conditions (Figures 2(a) and 2(b)). In addition, Gln-free medium obviously increased apoptosis in resistant cells but not in sensitive cells (Figure 3(a)), demonstrating that resistant cells require Gln to prevent programmed cell death. Taken together, these data suggested that resistant cell growth showed more dependence on Gln than sensitive cell growth.

### 3.2. Higher Basal Level of Autophagy Mediates ddp Resistance in ddp-Resistant Cells

**Figure 4:**
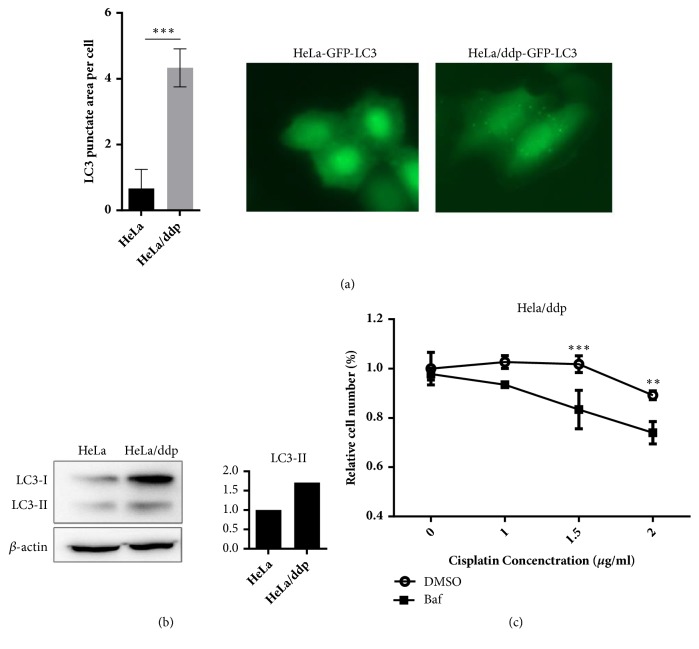
Ddp-resistant cells have a higher level of autophagy than ddp-sensitive cells, which mediates ddp resistance. (a) Aggregation of GFP-LC3 upon autophagosome formation in HeLa and HeLa/ddp cells. Quantifications of punctate areas per cell were analyzed in three different images containing more than 15 cells. (b) Western blot analysis of LC3-II protein expression in HeLa and HeLa/ddp cells; *β*-actin served as the loading control. (c) Relative proliferation of HeLa/ddp cells treated with increasing concentrations of ddp after autophagy inhibition by bafilomycin A1 (1 nM) for 72 h. The error bars represent the s.d. of triplicate wells of a representative experiment; *∗∗*P<0.01 and *∗∗∗*P<0.001.

Autophagy mediates ddp resistance in many cancers [5–11], and LC3 is the only protein that is especially correlated with autophagosomes, serving as a credible marker of autophagy in higher eukaryotes [18]. As demonstrated in Figure 4(b), LC3 protein expression was significantly increased in Hela/ddp cells, and consistent with the LC3 protein data, the number of puncta in GFP-LC3 Hela/ddp cells was also increased (Figure 4(a)). Furthermore, ddp resistance was reversed in HeLa/ddp cells when autophagy was inhibited by bafilomycin A_1_ (Baf, 1 nM) (Figure 4(c)), consistent with other studies, suggesting that increased autophagy contributes to ddp resistance in HeLa/ddp cells.

### 3.3. Gln Deprivation Triggers Apoptosis through the AMPK/ULK1 Pathway in ddp-Resistant Cells

**Figure 5:**
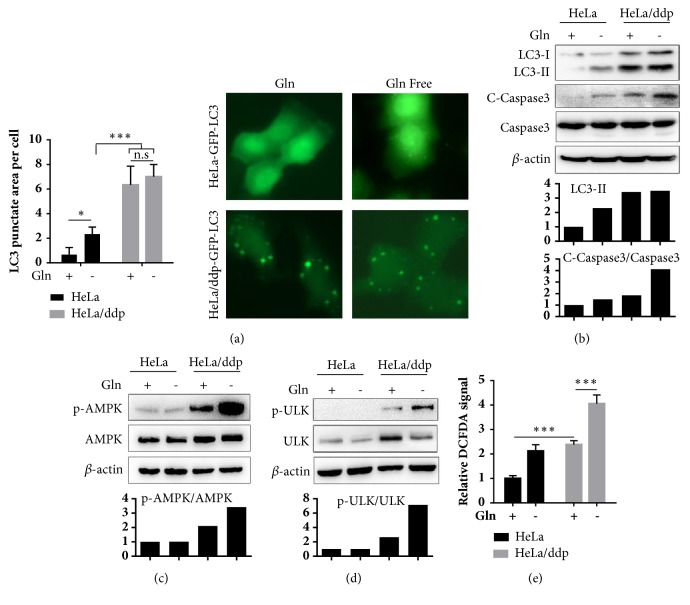
Gln deficiency induces apoptosis through the AMPK/ULK1 pathway in ddp-resistant cells. (a) Aggregation of GFP-LC3 upon autophagosome formation in HeLa and HeLa/ddp cells grown in the indicated medium for 24 h. Quantifications of punctate areas per cell were analyzed in three different images containing more than 15 cells. (b), (c), and (d) Western blot analysis of LC3-II, cleaved caspase-3, caspase-3, p-AMPK, AMPK, p-ULK1, and ULK1 protein expression in HeLa and HeLa/ddp cells grown in the indicated medium for 24 h using different antibodies; *β*-actin served as the loading control. (e) Relative ROS levels in HeLa and HeLa/ddp cells grown in the indicated medium for 24 h. The error bars represent the s.d. of triplicate wells of a representative experiment; *∗*P<0.05 and *∗∗∗*P<0.001.

Autophagy is known to be suppressed by glutaminolysis through the promotion of mammalian target of rapamycin complex 1 (MTORC1) activation and AMP-activated protein kinase (AMPK) inactivation [16]. Here, the levels of both LC3-II and punctate GFP-LC3 were slightly increased in ddp-sensitive cells but not in ddp-resistant cells in the Gln-free condition (Figures 5(a) and 5(b)), while the autophagy level in sensitive cells remained lower than that in ddp-resistant cells under Gln-free conditions. We also observed a significantly increased apoptosis rate in resistant cells in the Gln-free condition as demonstrated by the enhanced expression of cleaved caspase-3 (Figure 5(b)). Crosstalk between autophagy and apoptosis is highly context-dependent [19], as many cellular stress pathways can sequentially induce autophagy and apoptosis. Nonlethal stress often stimulates an autophagic response, and the apoptotic program is activated when stress exceeds a critical duration. These results suggest that ddp-resistant cells might face higher doses of stress in Gln-free conditions. AMPK is an energy sensor potentially activated in response to metabolic stress circumstances [20]. We found AMPK to be activated, and Gln deprivation could significantly enhance its activation in ddp-resistant cells but not in sensitive cells (Figure 5(c)). Meanwhile, we also observed that p-ULK1, which plays an essential role in the regulation of autophagy [21], was altered in a manner similar to that of p-AMPK in the same condition (Figure 5(d)). The ULK1 complex is activated via direct phosphorylation by AMPK, and this modification is critical for starvation-induced autophagy [22]. ULK1-triggered autophagy (particularly mitophagy) has also been confirmed to result in reactive oxygen species (ROS) overproduction, ultimately leading to apoptosis [23]. We next demonstrated that the level of ROS in ddp-resistant cells was higher than that in sensitive cells and significantly elevated in the Gln-free condition (Figure 5(e)), revealing that ULK1-triggered autophagy induced by Gln deficiency leads to apoptosis through ROS overproduction in ddp-resistant cells. Taken together, these data suggested that the same stress induced by Gln deficiency triggers apoptosis via the AMPK/ULK1 pathway in resistant cells because they have higher basal levels of autophagy and ROS, while this stress activates autophagy and helps the survival of sensitive cells.

### 3.4. Ddp-Resistant Cells Are Vulnerable to an Autophagy Inducer

**Figure 6:**
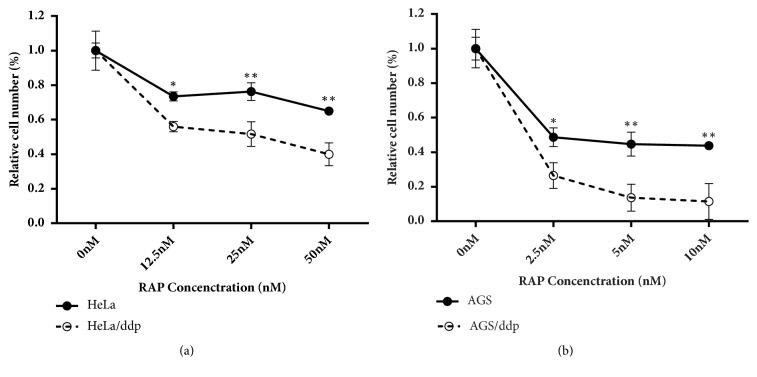
Ddp-resistant cells are more sensitive to rapamycin than ddp-sensitive cells. (a) Relative proliferation of HeLa and HeLa/ddp cells treated with increasing concentrations of rapamycin for 72 h. (b) Relative proliferation of AGS and AGS/ddp cells treated with increasing concentrations of rapamycin for 72 h. The error bars represent the s.d. of triplicate wells of a representative experiment; *∗*P<0.05 and *∗∗*P<0.01.

**Figure 7:**
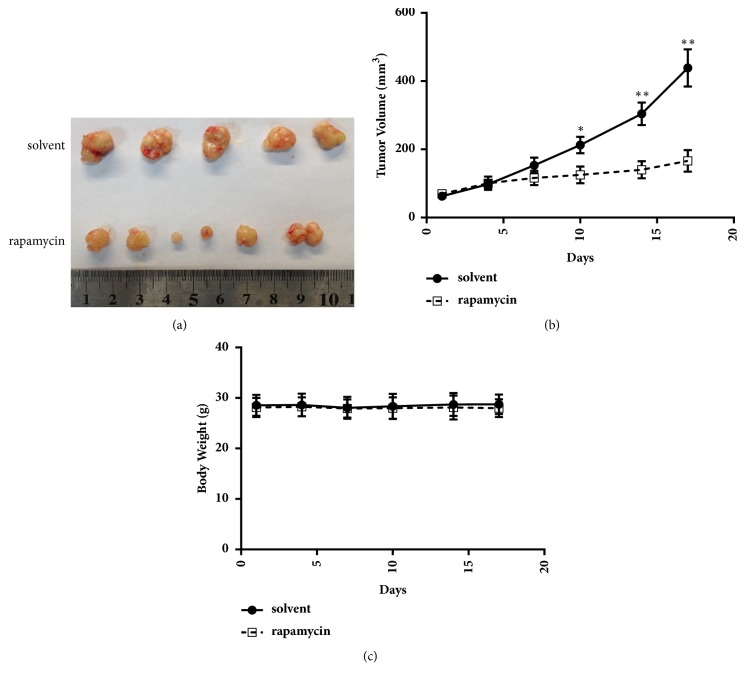
Rapamycin inhibits ddp-resistant tumor growth* in vivo*. (a) Resected tumors from each group (n=6 in the rapamycin group, n=5 in the solvent group). (b) Tumor growth kinetics; treatment was stopped after 14 days. Error bars indicate the s.e.m. (c) Body weights of mice in the two groups throughout the treatment period. The error bars indicate s.d. *∗*P<0.05 and *∗∗*P<0.01.

Because we demonstrated that the same stress induced protective autophagy in sensitive cells, while triggering apoptosis in resistant cells, we next investigated whether the autophagy inducer rapamycin could suppress the growth of ddp-resistant cells by further stimulating the autophagy signaling pathway. Rapamycin is a specific inhibitor of mTOR and can efficiently promote autophagy [24]. Our cell viability assay demonstrated that both Hela/ddp cells and AGS/ddp cells were more sensitive to rapamycin (Figures 6(a) and 6(b)). To further evaluate the implication of this special property on tumor treatment in ddp-resistant cells, we assessed the antitumor activity of rapamycin in a xenograft model. Tumors were established in Nu/Nu mice by injecting HeLa/ddp cells into their lower flanks and allowed to grow to approximately 50 mm^3^. The treatment group (n=6) received an i.p. injection of rapamycin (4 mg/kg) daily, whereas the control group (n=5) was injected with solvent, and treatment continued for 14 days. Rapamycin significantly reduced the growth of ddp-resistant tumors compared with that of control group tumors (Figures 7(a) and 7(b)). The mouse body weights remain unchanged during treatment, indicating that treatment with rapamycin caused no obvious animal toxicity (Figure 7(c)).

## 4. Discussion

Despite the fact that many studies have described mechanisms underlying the ddp-resistant phenotype in cancer cells, the high incidence of ddp chemoresistance remains the main limitation of its clinical usefulness. Thus, development of chemosensitization strategies has important clinical implications. Because autophagy is reinstated and increases the resistance of cancer cells to chemotherapy or radiotherapy, studies have focused mainly on how to reverse ddp resistance by inhibiting autophagy via different strategies. Our study yielded opposite results, as we found that further autophagy induction could trigger apoptosis and effectively suppress the growth of ddp-resistant cells.

Autophagy and apoptosis are two distinct self-destructive processes that determine the fate of damaged cytoplasmic organelles and whole cells, respectively. Generally, autophagy protects cells from stress and blocks the induction of apoptosis. However, in certain cases, autophagy or autophagy-relevant proteins may facilitate the activation of apoptosis [23, 25], and autophagy has also been shown to excessively degrade the cytoplasm, leading to “autophagic cell death” [19]. In our study, we revealed that although increased autophagy levels helped ddp-resistant cells tolerate treatment, it also made them more vulnerable to energy turbulence, especially in Gln-deficient conditions. In contrast, ddp-sensitive cells did not exhibit an elevated basal autophagy level, but autophagy levels increased slightly by Gln deficiency could promote the survival of sensitive cells during stress. Based on these phenomena, we tested whether ddp-resistant cells were more sensitive to autophagy induction than sensitive cells. Rapamycin, an autophagy inducer, could efficiently suppress the growth of resistant cells both* in vitro *and* in vivo*, which may have important implications in future strategies for treating ddp-resistant tumors.

In the present study, ddp-resistant cells had a significantly higher basal level of ULK1 than ddp-sensitive cells, and ULK1-triggered autophagy (particularly mitophagy) has been confirmed to result in ROS overproduction [23]. We also verified that ddp-resistant cells had increased autophagy and ROS levels. Although ROS at low levels could serve as critical signaling molecules in cell proliferation and survival, high ROS levels disrupt cellular processes, resulting in cell damage [26], and may also render cells vulnerable to further stresses [27]. We demonstrated that ddp-resistant cells were more sensitive to Gln deficiency, and ROS levels were also increased in the Gln-deficient condition. Therefore, whether ddp-resistant cells are vulnerable to other stresses, such as glucose deficiency, will be an interesting topic to pursue in future studies.

Taken together, our findings may have implications for future therapeutic approaches, as autophagy induction in ddp-resistant cells can potentially trigger ULK1-mediated apoptosis and provide novel mechanistic insights into reversal of the drug resistance effects of rapamycin. Therefore, rapamycin may conceivably represent a promising candidate for the treatment of ddp-resistant cancers.

## Data Availability

The data used to support the findings of this study are available from the corresponding author upon request.
